# Relationship Between Antihypertensive Medications and Cognitive Impairment: Part II. Review of Physiology and Animal Studies

**DOI:** 10.1007/s11906-016-0673-2

**Published:** 2016-08-04

**Authors:** Ruth Peters, Mattan Schuchman, Jean Peters, Michelle C. Carlson, Sevil Yasar

**Affiliations:** 1 School of Public Health, Imperial College London, London, SW7 2AZ UK; 2 Department of Medicine, Division of Geriatric Medicine & Gerontology, Johns Hopkins School of Medicine, Baltimore, MD 21224 USA; 3 School of Health and Related Research, University of Sheffield, Sheffield, UK; 4 Johns Hopkins Bloomberg School of Public Health, Department of Mental Health, Baltimore, MD 21205 USA

**Keywords:** Antihypertensive medication, Cognitive decline, Dementia, Alzheimer’s disease

## Abstract

**Purpose of Review:**

There is an established association between hypertension and increased risk of poor cognitive performance and dementia including Alzheimer’s disease; however, associations between antihypertensive medications (AHM) and dementia risk are less clear. An increased interest in AHM has resulted in expanding publications; however, none of the recent reviews provide comprehensive review. Our extensive review includes 24 mechanistic animal and human studies published over the last 5 years assessing relationship between AHM and cognitive function.

**Recent Findings:**

All classes of AHM showed similar result patterns in animal studies. The mechanism by which AHM exert their effect was extensively studied by evaluating well-established pathways of AD disease process, including amyloid beta (Aβ), vascular, oxidative stress and inflammation pathways, but only few studies evaluated the blood pressure lowering effect on the AD disease process.

**Summary:**

Methodological limitations of the studies prevent comprehensive conclusions prior to further work evaluating AHM in animals and larger human observational studies, and selecting those with promising results for future RCTs.

## Introduction

Alzheimer’s disease (AD), the most common cause of dementia [1], is characterized by extracellular amyloid beta (Aβ) deposition in the form of neuritic plaques, intracellular deposition of hyperphosphorylated microtubule-associated tau protein which culminates in synaptic loss, neuronal cell death, Aβ angiopathy, oxidative stress, and inflammatory processes resulting in cognitive impairment [2]. However, the exact mechanisms are still unclear.

There is a long established association between hypertension and increased risk of cognitive decline and AD in humans [3], but the potential association between antihypertensive treatment and reduced risk of AD has been harder to determine. Attempts to understand possible mechanisms have shifted attention toward the potential pleiotropic effects of the different classes of antihypertensive medication (AHM) and their potential impact on cognitive function [4, 5]. As a result, there are an increasing number of animal studies evaluating AHM, such as AHM acting through renin angiotensin system or altering calcium homeostasis.

This review aims to provide such an update in two parts. Part 1 provides an overview of the recent human observational and clinical trial literature, and part 2 reviews the recent physiological and animal work.

## Methods

### Search Strategy

The databases Embase, PsycINFO®, Medline, Medline in process, and other nonindexed citations and PubMed were searched from 2010 to February 2016 using the search terms: dementia or cognit* or mild cognitive impairment, and antihypertensives, or antihypertensive agents, or diuretic or diuretics or thiazide-like or calcium channel blocker or calcium channel blockers or calcium antagonist or angiotensin converting enzyme inhibitor or angiotensin-converting enzyme inhibitors or ACE inhibitors or angiotensin receptor blocker or angiotensin receptor blockers or angiotensin receptor blockers (ARB) or beta blocker or adrenergic beta-antagonists. Where review articles were identified, reference lists were searched for original research articles published within the last 5 years.

### Inclusion and Exclusion Criteria

Included animal or mechanistic studies required exposure to one of the antihypertensive classes of interest, calcium channel blockers (CCB), ARB, angiotensin converting enzyme inhibitors (ACE-I), beta blocker (BB), and diuretics, and to have a control or comparator group; however, a specific outcome measure was not required.

### Article Selection

Abstracts were double read by SY and MS. Discrepancies were resolved by discussion. Full text articles were double read by the same team and data extracted into standard tables, collated by antihypertensive class.

## Results

### Animal and Human Mechanism Studies

Searches retrieved 138 PubMed records and 522 records from Medline, PsycINFO® and Embase. Of these, seven were review articles [6–12], 19 articles presented results from animal studies exploring mechanisms [13–31], and there were five human studies of which one was an autopsy study [32], two human cerebrospinal fluid (CSF) studies [33, 34], one small RCT [35], and one in vivo human cell study [36].

The methodology of the 19 animal studies varied widely in the selection of animals and drugs used, length of treatment times, and outcomes. Animal models ranged from mouse models using wild-type mice [13, 23], aged Swiss mice [31], wild-type mice treated with icv amyloid beta 25–35 to be used as an AD mouse model [27], and transgenic (Tg) AD mice alone [17, 19, 21, 29, 30], to rat models such as Wistar [18, 20], Sprague-Dawley [16, 22], or spontaneously hypertensive rats (SHR) [14, 15, 24, 26, 28]. Regarding AHM used, 17 of the 19 studies used CCB, ACE-I, or ARB alone or in combination. Commercially available ACE-Is used were captopril [17, 18, 29], enalapril [27], imidapril [27], lisinopril [14], perindopril [26, 27], and trandolapril [19]. ARBs used included losartan [18, 19, 25], olmesartan [24], telmisartan [15, 22, 28], and valsartan [14]. The renin inhibitor aliskiren was used in one study [13]. CCBs used included azelnidipine [24], isradipine [20, 30], lercanidipine [14], nicardipine [14, 16, 19, 20, 30], nifedipine [30], nimodipine [16, 20, 22, 30], and a nonselective CCB flunarizine [31]. Other antihypertensives included BBs such as carvedilol and propranolol [19], diuretics such as amiloride and furosemide [19], and hydralazine [14, 19]. Experimental drugs such as angiotensin II [18], PD-123177 (angiotensin 2 receptor blocker) [20], and ICI 11,551 (a selective beta 2 receptor antagonist) [21] were also used. One study did not use antihypertensive medication [23]. One of the five identified human studies reported use of telmisartan and amlodipine [35], while four studies compared ARB users with other AHMs [32–34, 36]. Comparators used differed, as did the length of treatment times, which varied between 4 days and 15 months.

Outcome measures ranged from cognitive tests to biomarkers. Cognitive measures included water maze [15, 16, 19–21, 25, 26, 31], Y maze [13, 18], object recognition [21, 27], passive avoidance [18], open field [15, 22], and spontaneous alternation [27] tests. Locomotor function was assessed in five studies [22, 24, 26, 27, 31]. Numerous studies used serum, cerebrospinal fluid, or histopathological measures of amyloid beta and/or tau levels as their outcome [17, 19, 21, 23, 25, 29, 30]. Additional histopathological measures included pyramidal neurons in hippocampus [14], hippocampus morphology [16], and vascular pathology [24, 25]. Alteration in markers of oxidative stress [13, 18, 22, 24–26, 31], inflammation [13, 16, 22, 24, 26], apoptosis [20, 26], brain-derived neurotrophic factor (BDNF), and alpha tubulin levels [15] were also frequently used alone or in conjunction with cognitive measures. Some studies included measurement of various proteins of the renin angiotensin system (RAS) in the brain as their outcomes [13, 23, 25–27, 30]. Outcomes included infarct size [24, 31] and cerebral blood flow [13, 16, 24, 25] in studies evaluating the effect of antihypertensive medication in cerebral ischemia models. Surprisingly, only seven studies included blood pressure measurements as their outcome [13–15, 19, 24, 26, 28]. Human study outcomes also varied and included AD and vascular pathology in one autopsy study [32], amyloid and tau levels in cerebrospinal fluid (CSF) [33, 34], cognitive measures [36], and, in the small RCT, blood pressure measurements and cognitive outcomes [35].

### CCB

One human study reporting on the effect of CCB use found that of the 167 AHM users, only nifedipine users had significantly lower Aβ levels when compared to 107 matched AHM never users [36] (Table 1).

**Table 1:** Extraction table for mechanism studies: calcium channel blockers (CCB)

Author	Method: subjects	Methods: groups	Method: treatment	Method: treatment route	Methods: treatment time	Method: outcome	Method: statistic	Result
Daschil et al. [30]	-Tg (APP_SDI) mice -WT (C57BL/6N) mice	-Four groups	-Nicardipine -Nifedipine -Nimodipine		-4 weeks	-Aβ plaques in cortex -L-type calcium channel subunit expression in plaques -Angiogenesis in cortex slices after treating with L-type calcium channel blockers	Fisher *t* test	-Aβ plaques were detected in cortex of TgAPP mice, while none in WT mice -L-type calcium channel subunit expression seen was in plaques, but not around -L-type calcium channel inhibition with isradipine or nicardipine or nifedipine or nimodipine produced angiogenesis
Gholamipour-Badie et al. [20]	-Wistar rats	- Two groups	-Control -Aβ1–42 -Aβ1–42 + isradipine -Aβ1–42 + nimodipine	-Aβ1–42 injected into entorhinal cortex (EC) using stereotaxic surgery -CCBs were injected i.c.v.	-6 days	-Morris water maze test -Proteinase involved in calcium-dependent apoptosis (calpain 2, caspase 12, caspase 3)	ANOVA	-Aβ pretreated rats had delayed acquisition in memory tasks and this effect was reversed by higher dosages of isradipine, nimodipine -Calpain 2, Caspase 12 and 3 were increased in Aβ pretreated rats which was reversed isradipine, nimodipine
Gulati et al. [31]	-Aged Swiss mice	-*N* = 6	-Control -Sham surgery (no carotid occlusion) -Bilateral carotid occlusion and reperfusion -20 mg flunarizine 1 h before carotid occlusion and reperfusion -40 mg flunerazine 1 h before carotid occlusion and reperfusion -5 mg donepazil 1 h before carotid occlusion and reperfusion	-Flunarizine (nonselective CCB) -Donepezil (acetylcholinesterase inhibitor (AchE-I))		-Morris water maze test -Motor-in-coordination -Cerebral infarct size -Glutathione (GSH), total calcium and AchE activity in brain tissue	ANOVA	-Lower and higher dose of flunarizine, and donepezil decreased impairment of learning, memory, motor function, and infarct size -Stroke was associated with increased calcium and AchE activity and decreased GSH, which was reversed by flunarizine and donepezil
Justin et al. [22]	-Sprague Dawley rats	-*N* = 5	-Sham surgery (no carotid occlusion) -Bilateral carotid occlusion and reperfusion + carboxymethyl cellulose (CMC) -Bilateral carotid occlusion and reperfusion + telmisartan 5 mg/kg -Bilateral carotid occlusion and reperfusion + telmisartan 10 mg/kg -Bilateral carotid occlusion and reperfusion + telmisartan 5 mg/kg + nimodipine 5 mg/kg	-Telmisartan -Telmisartan + nimodipine	-9 days	-Day 2 neurological assessment -Day 7 behavioral assessment -Day 9 histopathological studies -Day 9 oxidative stress and inflammation markers	Logistic regression model	-Telmisartan pretreatment resulted in less neurologic deficit and locomotor function -Telmisartan increased glutamate, aspartate, and ATP levels in brain and decreased glutathione, nitric oxide levels -Telmisartan decreased the pro-inflammatory cytokine (IL-1β, IL-6, TNF-α), lipid peroxide and nitric oxide levels; and increased anti-inflammatory cytokine IL-10 level -Best results in all was when telmisartan was given in combination with nimodipine
Lovell et al. [36]	-Humans (case control study, *N* = 1100, >60 years old, mild dementia)	-*N* = 274	-*N* = 32 CCB users with *N* = 31 matched nonusers -*N* = 13 ACE-I users with *N* = 13 matched nonusers -*N* = 22 BB user with *N* = 21 matched nonusers	-Antihypertensives -ACE-I -CCB -BB		-Progression to dementia -Aβ1–42 production in cell culture pretreated with nifedipine, amlodipine, diltiazem, felodipine, isradipine, nicardipine, nimodipine, nisoldipine	Regression model	-CCB users had less decline than nonusers, and it decreased effect of ApoE presence -Nifedipine decreased most significantly by 40 % Aβ1–42
Omote et al. [24]	-Spontaneously hypertensive rats (SHR)	-*N* = 6	-Control was treated with carboxymethyl cellulose (CMC) -Olmesartan low dose 2 mg/kg -Olmesartan high dose 10 mg/kg -Azelnidipne low dose 2 mg/kg -Azelnidipne high dose 10 mg/kg -Combination olmesartan 1 mg/kg and azelnidipine 1 mg/kg	-po	-14 days	-Blood pressure (BP) -Pulse rate -Bodyweight -Regional cerebral blood flow (rCBF) -Serum triglyceride, LDL, HDL -Oxidative stress markers -Inflammatory markers -Neurovascular units -Infarct volume -Motor coordination and balance	ANOVA	-BP, pulse, decreased while body weight, rCBF remained stable in all treatment groups compared to control group -Infarct volume decreased with olmesartan and azelnidipine treated group, and it was better than low-dose monotherapy; however, high-dose azelnidipine (better BP reduction) was better than high-dose olmesartan -All treatments in dose response matter reduced oxidative markers, inflammatory markers, and preserved neurovascular unit
Sakurai-Yamashita et al. [14]	-Spontaneously hypertensive rats (SHR)	-*N* = 6	-Control with carotid occlusion -Carotid occlusion and lercanidipine -Carotid occlusion with nicardipine -Carotid occlusion with lisinopril -Carotid occlusion with valsartan -Carotid occlusion with hydralazine	-Lercanidipine and nicardipine po in diet -Valsartan osmotic pump -Lisinopril osmotic pump -Hydralazine osmotic pump	-14 days	-Delayed neuronal death of pyramidal neurons in hippocampus -Blood pressure	ANOVA	-Blood pressure was reduced in all treatment groups but only lercanidipine protected against neuronal death, while other treatments did not provide protection
Wang et al. [19]	-Tg2576 mice		-1600 FDA approved drugs were screened for Aβ regulating effect: 184 drugs lowered Aβ by >30 % and 26 drugs increased Aβ levels by >30 % -short term treatment: propranolol, carvedilol, nicardipine, losartan, amiloride, hydralazine, furosemide, trandalopril -long term treatment: nicardipine, propranolol	-Drinking water	-1 month -Selected group for chronic treatment: 6 months	-Blood pressure measurement -Total Aβ1–40 or Aβ1–42 in brain and plasma after short term treatment -Morris water maze test after long-term treatment	ANOVA	-Short-term use: propranolol, losartan significantly reduced blood pressure by 20 %; propranolol, nicardipine, carvedilol reduced significantly Aβ1–42 and 1–40 by 40 % in the brain and plasma, losartan reduced Aβ1–42 but not 1–40 -Furosemide and trandalopril significantly reduced Aβ without affecting BP -long term treatment with propranolol and nicardipine did not improve cognition, but decreased Aβ in brain but not plasma
Zhang et al. [16]	-Sprague-Dawley rats	-*N* = 4	-Sham surgery -Focal cerebral ischemia (induced by 15 min treatment with intraluminal filament) -Vascular dementia (bilateral carotid artery occlusion and reperfusion) -Vascular dementia (bilateral carotid artery occlusion and reperfusion) pretreated with nimodipine 20 mg/kg	-Via gastric perfusion	-4 days	-Morris water maze test -Brain MRI PWI -Hippocampal levels of nuclear factor kB (NF-kB), tumor necrosis factor α (TNF-α), interleukin 1β (IL-1β -Hippocampus nerve cell morphology	ANOVA	-Vascular dementia group pretreated with nimodipine performed better on learning, had better regional cerebral blood flow, and had lower levels of NF-kB, TNF-α, IL-1β; hippocampus cell morphology was almost normal in this group similar to normal control and focal ischemia group

Eight animal studies reported results of treatment with CCB alone [14, 16, 19, 20, 30, 31] or in combination with ARB [22, 23]. Azelnidipine decreased blood pressure, infarct size, and also reduced markers of oxidative stress and inflammation [24]. The nonselective CCB flunarizine reversed impairment in learning, memory, and motor function after cerebral ischemia and reversed cerebral ischemia-associated decrease in anti-oxidative stress markers [31]. Isradipine increased angiogenesis [30] and improved memory acquisition [20]. Lercanidipine decreased blood pressure and protected against neuronal death [14]. Nicardipine reduced Aβ1–42 and Aβ1–40 in the brain [19] and increased angiogenesis [30], but did not improve cognition [19]. Nifedipine increased angiogenesis [30]. Nimodipine improved regional cerebral blood flow and protected hippocampal morphology [16], reduced inflammatory markers [16], increased angiogenesis [30], improved memory acquisition [20], and prevented learning impairment in animals with cerebral ischemia [16] (Table 1).

### ACE-I

The most extensively studied ACE-I was captopril, which was associated with genetic upregulation of proteins associated with neuronal function and membranes [17], reduced Aβ burden in the brain [17], decreased conversion of Aβ1–43 to Aβ1–42 [28], increased anti-oxidative stress markers [18], decreased oxidative stress markers [17], and better performance on learning and memory tasks [18]. Captopril treatment also inhibited ACE activity and decreased angiotensin II levels [17, 29]. Lisinopril did not protect against neuronal death even with significant blood pressure reduction [14]. Perindopril and enalapril inhibited plasma ACE activity by 90 % but only perindopril inhibited brain ACE activity by 50 % [27]. Perindopril decreased blood angiotensin II levels [26] and also levels of oxidative stress markers [26]. Perindopril improved memory function [26]. Trandalopril treatment reduced Aβ burden in the brain [19] (Table 2).

**Table 2:** Extraction table for mechanism studies: angiotensin receptor blocker (ARB), angiotensin converting enzyme inhibitor (ACE-I), and diuretic

Author	Method: subjects	Methods: groups	Method: treatment	Method: treatment route	Methods: treatment time	Method: outcome	Method: statistic	Result
AbdAlla et al. [17]	-Aged Tg2576 mice	-*N* = 2	-Control -Captopril		-6 months	-Aβ plaque load in hippocampus	ANOVA	-Captopril treated Tg mice had significantly lower Aβ, upregulated genes associated with neuronal membrane and neuronal process, reduced oxidative stress markers, reduced amyloidogenic process of APP, and decreased ACE and angiotensin II levels -This effect is most likely direct effect of the centrally acting captorpil (not observed in enalapril which only acts peripherally)
Bild et al. [18]	-Wistar rats	-*N* = 5	-Control (saline) -Angiotenisn II -Captorpil -Losartan -PD-123177	-i.c.v.	-7 days	-Y-maze task -Step through passive avoidance task -Superoxide dismutase (SOD) activity in hippocampus -Glutathione peroxidase (GPX) activity in hippocampus -Malondialdehyde (MDA) levels in hippocampus	ANOVA	-On Y maze task angiotensin II-treated animals preformed worse and captoril, losartan and PD 123177 treated animals did significantly better when compared to control group -In avoidance task angiotensin II-treated animals preformed worse and losartantreated animals performed better when compared to control group -SOD, GPX, and MDA activity was decreased in angiotensin II-treated rats and increased in captopril-treated rats, when compared to control group
Dong et al. [13]	-WT mice (C57BL/6 J)	-*N* = 3	-Sham surgery -Bilateral carotid occlusion and reperfusion + vehicle -Bilateral carotid occlusion and reperfusion + aliskiren 2.5 mg/kg	-Mini pump	-35 days	-Blood pressure -Cerebral blood flow -Y-maze test -Superoxide dismutase (SOD) -Tumor necrosis factor α (TNF α) -Brain angiotensin and renin expression	ANOVA	-Aliskerin did not alter blood pressure or cerebral blood flow -Aliskerin-treated animals did better on Y-maze task -Aliskerin treatment did not alter SOD levels but did TNF α -Aliskerin treatment decreased renin and angiotensin expression -Aliskerin treatment decreased white matter lesions in brain
Goel et al. [26]	-Wistar rats -Spontaneously hypertensive rats (SHR)	-*N* = 8	-Wistar rats treated with vehicle -Wistar rats treated with lipopolysaccharide (LPS) 25 μg to induce inflammation -Wistar rats treated with LPS 50 μg -Wistar rats treated with LPS 50 μg + perindopril 0.1 mg/kg -Wistar rats treated with perindopril 0.1 mg/kg -SHR treated with venicle -SHR treated with treated with LPS 25 μg -SHR treated with treated with LPS 25 μg + perindopril 0.1 mg/kg -SHR treated with perindopril 0.1 mg/kg	-i.c.v. LPS -po perindopril	-15 days	-Blood pressure -Morris water maze test -Spontaneous locomotor activity (SLA) -Brain tissue (cortex, hippocampus) ACE activity, ACE gene expression, angiotensin II level, cytokine level, reactive oxidative species (ROS), nitrate, caspase 3 activity, TNF α, NF-kB, IL-10,	ANOVA	-LPS caused impaired memory in SHR and in normal rats which was reversed by perindopril -In SHR ACE activity and gene expression, angiotensin II level, oxidative stress, and inflammatory markers were increased -LPS caused further increase in ACE activity and gene expression, angiotensin II level, oxidative stress, and inflammatory markers, which was decreased by perindopril
Hajjar et al. [32]	-*N* = 890 participants with hypertension and autopsy available	-*N* = 3	-*N* = 133 have used ARB -*N* = 577 have used other antihypertensive medication -*N* = 180 have not used antihypertensive medication	-po		-AD pathology (CERAD, ADRDA/Khachaturian, Pathologic diagnosis) -Vascular pathology (large artery infarcts, microinfarcts, hemorrhage, atherosclerosis, arteriosclerosis)	Logistic regression analysis	-ARB users when compared to other and no antihypertensive medication users had significantly less AD pathology ( 50 %reduction) -ARB use was associated with higher vascular pathology
Hajjar and Levey 2015 [33]	-*N* = 319 participants with CSF available	-*N* = 2	-*N* = 26 have used ARB -*N* = 293 have used other antihypertensive medication	-po	-3 years	-ARB use and longitudinal tau decline in people with dementia, MCI, and normal controls	Mixed model	-ARB use was associated with decreased levels of tau and p-tau in people with MCI when compared to other antihypertensive medication users
Justin et al. [22]	-Sprague Dawley rats	-*N* = 5	-Sham surgery (no carotid occlusion) -Bilateral carotid occlusion and reperfusion + carboxymethyl cellulose (CMC) -Bilateral carotid occlusion and reperfusion + telmisartan 5 mg/kg -Bilateral carotid occlusion and reperfusion + telmisartan 10 mg/kg -Bilateral carotid occlusion and reperfusion + telmisartan 5 mg/kg + nimodipine 5 mg/kg	-po	-9 days	-Day 2 neurological assessment -Day 7 behavioral assessment -Day 9 histopathological studies -Day 9 oxidative stress and inflammation markers	Logistic regression model	-Telmisartan pretreatment resulted in less neurologic deficit and locomotor function -Telmisartan increased glutamate, aspartate, and ATP levels in brain and decreased glutathione, nitric oxide levels -Telmisartan decreased the pro-inflammatory cytokine (IL-1β, IL-6, TNF-α), lipid peroxide and nitric oxide levels; and increased anti-inflammatory cytokine IL-10 level -Best results in all test was when telmisartan was given in combination with nimodipine
Kishi et al. [15]	-Spontaneously hypertensive rats (SHR)	-*N* = 5	-Vehicle -Telmisartan 1 mg/kg -Telmisartan 1 mg/kg + GW9662 1 mg/kg (a PPAR gamma antagonist) -GW9662 1 mg/kg -Telmisartan 1 mg/kg + ANA-12 0.5 mg/kg)	-po	-4 weeks	-Blood pressure and heart rate -Morris water test -Hippocampus BDNF level and α-tubulin expression	ANOVA	-Blood pressure and heart rate was not changed in any of the treatment groups -Animals treated with telmisartan alone or with GW9662 or with ANA-12 had higher BDNF levels in hippocampus than the comparison groups -Animals treated with telmisartan alone or with GW9662 performed better on Morris water test than the comparison groups
Kume et al. [35]	-20 patients with hypertension and AD	*N* = 2	-*N* = 10 Telmisartan 40-80 mg daily -*N* = 10 Amlodipine 5-10 mg daily	-po	-6 months	-Blood pressure -Heart rate -Cognitive test (MMSE, ADAS-Jcog, logical memory) -Regional cerebral blood flow (rCBF) measured by SPECT	ANCOVA	-Systolic blood pressure was better in both groups -rCBF improved significantly in areas of frontal, parietal, occipital lobe in telmisartan users- no improvement in cognition
Liu et al. [23]	-Wild mice (C57BL/6J) -Humans with AD and normal controls					-ACE2 (which transforms angiotensin 1–10 to angiotensin 1–9) activity (mice) -Aβ levels (Aβ43, Aβ42, Aβ40) -Angiotensin II levels (humans)	Mann-Whitney *U* test	-ACE2 converted Aβ43 to Aβ42 -ACE converted Aβ43 to Aβ40 -ACE2 activity was decreased in human serum -Serum angiotensin levels were similar in people with AD and normal controls
Nation et al. [34]	-*N* = 871 stroke and dementia free people with available CSF	-*N* = 3	-*N* = 90 have used ARB -*N* = 343 have used other antihypertensive medication -*N* = 438 have used no antihypertensive medication	-po	-24 months	-CSF Aβ1–42, p-tau levels -progression to dementia	ANCOVA	-Older people had lower CSF Aβ1–42 -ARB users had higher levels of CSF Aβ1–42 and lower levels of CSF p-tau then the other groups -ARB users were less likely to progress to dementia
Omote et al. [24]	-Spontaneously hypertensive rats (SHR)	-*N* = 6	-Control group was treated with carboxymethyl cellulose (CMC) -Olmesartan low dose 2 mg/kg -Olmesartan high dose 10 mg/kg -Azelnidipne low dose 2 mg/kg -Azelnidipne high dose 10 mg/kg -Combination olmesartan 1 mg/kg and azelnidipine 1 mg/kg	-po	-14 days	-Blood pressure (BP) -Pulse -Body weight -Regional cerebral blood flow (rCBF) -Serum triglyceride, LDL, HDL -Oxidative stress markers -Inflammatory markers -Neurovascular units -Infarct volume -Motor coordination and balance	ANOVA	-BP and pulse decreased while body weight and rCBF remained stable in all treatment groups compared to control group -Infarct volume decreased in olmisartan and azelnidipine-treated group, and it was more effective than low-dose monotherapy; however, high-dose azelnidipine (better BP reduction) was more effective than high-dose olmesartan -All treatments reduced oxidative markers, inflammatory markers, and preserved neurovascular unit in a dose-reponse matter
Ongali et al. [25]	-TgAPP mice -Wild-type mice	-*N* = 4	-Wild-type treated with vehicle -Wild-type treated with losartan 1 and later 10 mg/kg -TgAPP treated with vehicle -TgAPP treated with losartan 1 and later 10 mg/kg	-po	-3 months (old) and 10 months (young)	-Morris water maze test -Cerebral blood flow CBF) -Glucose FDG-PET -Blood pressure -Vascular reactivity in brain tissue -AD neuropathology -SOD levels in brain -Angiotensin 1 and 4 receptors (ANG1R, ANG4R)	ANOVA	-In old TgAPP mice losartan did not improve learning but improved memory acquisition and recall -In old TgAPP SOD, ANG1R, and ANG4R levels were increased which was reduced with Losartan -Losartan increased CBF i cerebral glucose uptake, and cerebrovascular responsivenessin old TgAPP mice -Losartan did not decrease AD pathology (Aβ1–42)
Wang et al. [19]	-Tg2576 mice		-1600 FDA approved drugs were screened for Aβ regulating effect: 184 drugs lowered Aβ by >30 % and 26 drugs increased Aβ levels by >30 % -Short-term treatment: propranolol, carvedilol, nicardipine, losartan, amiloride, hydralazine, furosemide, trandalopril -Long-term treatment: nicardipine, propranolol	-Drinking water	-1 month -Selected group for chronic treatment: 6 months	-Blood pressure measurement -Total Aβ1–40 or Aβ1–42 in the brain and plasma after short-term treatment -Morris water maze test after long-term treatment	ANOVA	-Short-term use: propranolol, losartan significantly reduced blood pressure by 20 %; propranolol, nicardipine, carvedilol reduced significantly Aβ1–42 and 1–40 by 40 % in brain and plasma, losartan reduced Aβ1–42 but not 1–40 -Furosemide and trandalopril significantly reduced Aβ without affecting BP -Long-term treatment with propranolol and nicardipine did not improve cognition but decreased Aβ in brain but not plasma
Yamada et al. [27]	-Wild-type mouse treated i.c.v Aβ25–35 (AD mouse model)	-*N* = 3	-Perindopril (0.1, 0.3, 1.0 mg/kg) -Imidapril (0.3, 1.0, 3.0 mg/kg) -Enalapril (1.0, 3.0, 10 mg/kg)	-po	-5 days	-Spontaneous alteration test (SAT) -Object recognition test -Spontaneous locomotor activities -Anxiety-related behavior in elevated plus maze -ACE activity in brain and plasma	ANOVA	-Perindopril in all dosages improved working memory (measured by SAT), object recognition -Plasma ACE was inhibited perindopril 1 mg/kg, imidapril 3 mg/kg, 10 mg/kg enalapril by 90 % -Brain SACE was inhibited by 50 % by 1 mg/kg perindopril but was less in other ACE-Is
Zhai et al. [28]	-Wistar rats -Spontaneously hypertensive rats (SHR)	-*N* = 4	-Wistar rats -SHR treated with Vehicle -SHR treated with Telmisartan 0.3 mg/kg SHR -SHR treated with Telmisartan 3 mg/kg SHR	-po	-3, 9, and 15 months	-Blood pressure -LDL receptor, ApoE expression in the cortex and hippocampus	ANOVA	-In the cortex and hippocampus of SHR ApoE expression and LDL receptors was increased at all ages but was significantly reduced in both doses of telmisartan -At low dose, blood pressure remained unchanged
Zou et al. [29]	-Tg2576 mice -APP J20 mice	-*N* = 3	-Tg2576 mice treated with captopril 0.25 mg/kg -Tg2576 mice treated with vehicle -TgAPP J20 mice	-po	-11 months	-Aβ levels in brain (Aβ1–40, Aβ1–42, Aβ1–43) -ACE activity in brain	Student’s *t* test Spearman’s rank test	-In TgAPP mouse Aβ1–43 occurs before Aβ1–40 and Aβ1–42 -ACE converted Aβ1–43 to Aβ1–40 -Captopril pretreatment decreased ACE activity by 26 % and increased Aβ1–43 deposition -In people with AD serum, Aβ1–43 level is higher and CSF level is lower when compared to normal control

### ARB

Losartan decreased angiotensin 1 and 4 receptor levels in the brain [25] and improved cerebral blood flow [25]. In one study, it decreased Aβ1–42 [19], while in another, it did not alter Aβ1–42 in the brain [25]. Treatment with losartan also resulted in better performance on learning and memory tasks [18, 25]. Telmisartan improved cerebral blood flow in humans [35], reduced neurologic deficits and improved locomotor function after cerebral ischemia [22, 35], reduced inflammatory and oxidative stress markers [22], reduced low-density receptors and apolipoprotein E expression in the brain [28], and increased BDNF levels in the hippocampus [15]. Treatment with telmisartan resulted in better performance on learning and memory tasks in animals [15]; however, there was no improvement in memory in people [35]. Olmisartan did not reduce blood pressure but reduced infarct size in cerebral ischemia and inflammatory markers [24]. Valsartan reduced blood pressure but did not protect against neuronal death [14] (Table 2).

ARBs were studied as a class in human studies. One brain autopsy study showed that ARB use was associated with significantly lower AD pathology, while no alteration of vascular pathology was observed when compared to other or no antihypertensive medication users [32]. Additionally, it was found that ARB use in people with normal cognition or mild cognitive impairment (MCI) was associated with lower levels of tau and phosphorylated tau [32, 34] and higher levels of Aβ1–42 in cerebrospinal fluid [34], and with decreased risk of dementia [34] when compared to other antihypertensive medication users (Table 2).

### Diuretics

Only one animal study evaluated a diuretic, furosemide, and found that it reduced brain Aβ1–42 without affecting blood pressure [19].

### BBs

Two animal studies reported on the effect of BB use (Table 3). Treatment with nonselective beta adrenergic receptor blockers, carvedilol and propranolol, resulted in decreased brain Aβ1–40 and Aβ1–42 levels; however, this did not translate into improved cognition [19]. Carvedilol reduced Aβ1–42 in the brain without affecting blood pressure [19]. In contrast, treatment with a selective beta 2 adrenergic receptor (β2AR) antagonist resulted in significantly worse working memory and increased amyloid plaque burden, Aβ1–42 levels, tau phosphorylation, and accumulation in the hippocampus, suggesting involvement of β2ARs in the amyloid pathway and in cognitive function [21].

**Table 3:** Extraction table for mechanism studies: beta blockers

Author	Method: subjects	Methods: groups	Method: treatment	Method: treatment route	Methods: treatment time	Method: outcome	Method: statistic	Result
Branca et al. [21]	-3xTg-AD mice -Non-Tg mice	-Four groups	-ICI 11,551 (selective β2-adrenergic receptor antagonist) -NaCl	-Intraperitoneal injections daily 1 mg/kg	-6 weeks	-Morris water maze test -Novel object recognition test -Aβ42 and tau levels in hippocampus -Proteosome activity assay	ANOVA	-Significantly better performance on Morris water maze in non-Tg-AD mice treated with NaCl, while worse in non-Tg mice treated with ICI, and non-Tg mice treated with NaCl, and worse when treated with ICI -Aβ42 and tau levels in hippocampus of ICI-treated 3xTg-AD mice was significantly higher than when treated with NaCl suggesting increased Aβ production

## Discussion

The importance of dementia as a clinical and public health issue is rapidly increasing as the population ages [37]. Thus, identifying new and effective approaches to prevention or treatment is critical. Due to the lengthy process of developing new medications, there has been a recent surge in interest toward re-purposing currently available medications for the treatment of AD, including AHM. In this paper, we provide an extensive review of 24 mechanistic animal and human studies published over the last 5 years assessing the relationship between AHM and cognitive function.

Previous studies have shown a possible protective effect of certain AHM against AD risk [1], and it has been suggested that this protective effect is independent of, or in addition to, the blood pressure lowering effect [4, 5]. It is therefore not surprising that the mechanistic studies have focused on evaluating effects of AHM on well-established pathways in the AD disease process, including Aβ, vascular, oxidative stress, and inflammation pathways [2].

Of the six CCBs, nimodipine has been the most widely studied, and it was associated with angiogenesis and neuroprotection in the hippocampus, reduced inflammation, and improved cognitive function, but not with improved cerebral blood flow. Flunarizine and isradipine also improved cognition and had some effect on some of the above mentioned pathways.

Of the five ACE-Is studied, most studies evaluated effects of captopril and perindopril. Captopril was associated with neuroprotection [17], reduced Aβ burden in the brain [17, 29], decreased oxidative stress [17, 18], and better cognitive performance [18]. This effect was mediated by alteration of ACE activity and angiotensin II levels in the brain [17, 29]. Perindopril was associated with decreased oxidative stress [26] and improved cognitive function [26] and was shown to inhibit ACE activity in both blood and the brain ACE [27]. These findings suggest the beneficial effect of ACEs when crossing the blood-brain barrier; however, a previous observational study by Sink et al. did not support this hypothesis [38].

Of the four ARBs studied, losartan and telmisartan were examined in detail. Losartan use was associated with improved cerebral blood flow [25]. Yet, its effect on Aβ1–42 was equivocal with one study showing decreased levels [19], while another unchanged levels of Aβ1–42 [25]. Treatment with losartan also resulted in better performance on learning and memory tasks [18, 25]. These findings suggest beneficial effect via vascular rather than amyloid pathways, which is supported by its angiotensin 1 and 4 receptor lowering effect in the brain [25]. The other medication evaluated in detail was telmisartan, which, similar to losartan, was associated with improved cerebral blood flow in humans [35], reduced inflammation, oxidative stress [22], and markers of brain lipid metabolism [28]. Telmisartan also improved cognitive performance in animals [15], however, not in humans [35]. This negative finding in humans was replicated in a large multinational double-blind randomized placebo controlled trial, TRANSCEND, comparing ARB (telmisartan) use to placebo [3].

Previous animal studies and also RCTs with AHM have shown that blood pressure reduction, particularly in close proximity to development of cognitive impairment, does not alter dementia risk. Additionally, it is possible that treatment comes too late to mitigate the injury related to chronic exposure, suggesting an earlier window of benefit after which neural damage is hard to remediate. Thus, other mechanisms involved in AD development should to be explored. Medications explored in mechanistic studies have been different agents to those used in RCTs.

## Conclusions

Similar to human observational studies and RCTs, different classes of AHM show similar result patterns in animal studies.

Inconsistencies in the sources of evidence from the use of different animal types, ages, treatment times, and outcome measures limit the possibility of drawing firmer conclusions. Similar to observational studies, the relative lack of information on blood pressure levels is a major limitation. These limitations restrict our ability to draw wider ranging conclusions about use of specific antihypertensive classes, subclasses, or individual drugs. However, AHM that have had promising results in animals and larger human observational studies should be selected for future RCTs.
